# EXPLORING THE IMPACT OF GUT MICROBIOTA MODULATION ON COLORECTAL CANCER THERAPY: A BIBLIOMETRIC ANALYSIS OF PROBIOTIC AND PREBIOTIC INTERVENTIONS

**DOI:** 10.1590/S0004-2803.24612025-087

**Published:** 2026-03-23

**Authors:** Elisha Apatewen AKANBONG, Ali ŞENOL, Özkan DURU, Erva ESER, Haceli ÖCAL, Miyase ÇINAR

**Affiliations:** 1 Kırıkkale University, Faculty of Veterinary Medicine, Department of Biochemistry, Kırıkkale, Turkey.; 2 Kırıkkale University, Faculty of Veterinary Medicine, Department of Biostatistic, Kırıkkale, Turkey.

**Keywords:** Gut microbiota, colon cancer, colorectal cancer, gut microbiota modification, Microbiota intestinal, câncer de colon, câncer colorretal, modificação da microbiota intestinal

## Abstract

**Objective:**

This study is a bibliometric analysis of scientific publications reporting the beneficial effects of gut microbiota modulation by probiotics and/or prebiotics on colorectal cancer.

**Methods:**

This study adhered to the PRISMA guidelines and focused on English peer-reviewed research articles published between 2020 and 2025, as indexed in Google Scholar and PubMed. Search terms included “Colorectal Cancer”, “Colon Cancer”, “Colorectal Carcinoma”, “Colon Carcinoma” and “Gut Microbiota”. A total of 116 studies were selected and manually reviewed, taking into account the inclusion and exclusion criteria.

**Results:**

The bibliometric analysis of 37 studies revealed that China leads research efforts, primarily focusing on prebiotics (70.3%), such as dietary fibres, while probiotic-focused studies are limited (24.3%) due to practical challenges. Although the combined use of prebiotics and probiotics (2.7%) is theoretically beneficial, their practical application remains complicated. The trend in the literature aligns with a growing interest in traditional medicine and functional foods, but annual publication rates have declined, with only three papers from 2025 (30.8%). Despite recognising microbial diversity, the number of probiotic-based studies is low, with *Clostridium butyricum* being the most prevalent species.

**Conclusion:**

Overall, this analysis underscores the critical role of gut microbiota in CRC treatment and the potential of natural compounds, while highlighting the need for further research. Further research is essential to deepen our understanding of gut microbiota dynamics and optimise therapeutic approaches for patients with CRC.

## HIGHLIGHTS

• Dominant Contributor: China leads the field of research on gut microbiota modulation and colorectal cancer, contributing 22 publications, which constitute 59.5% of the total dataset.

• Prebiotic Focus: A substantial majority of the studies, 26 out of 37 (70.3%), emphasise the exclusive use of prebiotics for modulating gut microbiota.

• Probiotic Utilisation: Nine studies (24.3%) were dedicated solely to the application of probiotics, highlighting their role in gut health.

• Limited Synbiotic Research: A mere 1 study (2.7%) investigated a synbiotic approach, indicating a gap in research combining both probiotics and prebiotics.

• Overall Research Trends: The analysis reveals a significant emphasis on prebiotics over probiotics, underscoring the need for further investigation into integrated approaches for gut microbiota modulation in colorectal cancer. These highlights succinctly capture key insights from the bibliometric analysis and underscore the current research landscape in this area.

## RESUMO

**Contexto e Objetivo:**

Este estudo é uma análise bibliométrica de publicações científicas que relatam os efeitos benéficos da modulação da microbiota intestinal por probióticos e/ou prebióticos no câncer colorretal.

**Métodos:**

Este estudo seguiu as diretrizes PRISMA e se concentrou em artigos de pesquisa revisados por pares em inglês, publicados entre 2020 e 2025, indexados no Google Acadêmico e PubMed. Os termos de busca incluíram “Câncer Colorretal”, “Câncer de Cólon”, “Carcinoma Colorretal”, “Carcinoma de Cólon” e “Microbiota Intestinal”. Um total de 116 estudos foram selecionados e revisados manualmente, levando em consideração os critérios de inclusão e exclusão.

**Resultados:**

A análise bibliométrica de 37 estudos revelou que a China lidera os esforços de pesquisa, com foco principal em prebióticos (70,3%), como fibras alimentares, enquanto os estudos focados em probióticos são limitados (24,3%) devido a desafios práticos. Embora o uso combinado de prebióticos e probióticos (2,7%) seja teoricamente benéfico, sua aplicação prática permanece complexa. A tendência na literatura alinha-se com o crescente interesse em medicina tradicional e alimentos funcionais, mas as taxas anuais de publicação diminuíram, com apenas três artigos publicados em 2025 (30,8%). Apesar do reconhecimento da diversidade microbiana, o número de estudos baseados em probióticos é baixo, sendo Clostridium butyricum a espécie mais prevalente.

**Conclusão:**

De modo geral, esta análise ressalta o papel crítico da microbiota intestinal no tratamento do CCR e o potencial dos compostos naturais, ao mesmo tempo em que destaca a necessidade de mais pesquisas. Mais pesquisas são essenciais para aprofundar nossa compreensão da dinâmica da microbiota intestinal e otimizar as abordagens terapêuticas para pacientes com CCR.

## INTRODUCTION

Colorectal cancer (CRC) is a common cancer that poses a major global health burden. Globally, CRC is ranked third in terms of incidence and second in terms of mortality, accounting for 1.8 million new cases and 881,000 deaths in 2018. Its incidence is predicted to increase to 2.2 million new cases and 1.1 million deaths worldwide by 2030^1^. CRC, with over 1 million new cases diagnosed annually, is the third most prevalent type of cancer in men and the second most prevalent type of cancer in women and the primary cause of cancer-related deaths^2^. CRC has complex causes involving both genetic and environmental factors^1^. However, there has been a longstanding interest in the role of gut microbiota in CRC development and progression. This curiosity is partly due to the high microbial load in the colon and the potential involvement of the gut microbiota in carcinogenesis.

Although microbiota colonise body surfaces, including the oral cavity, vagina, skin, and fluids, they predominantly inhabit the gastrointestinal tract (GIT)^3^. After being acquired through vertical transmission (through birth), the gut microbiota becomes prone to changes by environmental (external) factors such as lifestyle, drug treatment, and diet (nutrition) and co-evolves concomitantly with the host throughout life^4^. A disturbance in the health and stability of this ecosystem (microbial balance) causes dysbiosis, which subsequently leads to a multitude of pathological states, including gastrointestinal disorders, cardiovascular, respiratory, neurological, and metabolic conditions, stomach cancer, and CRC^5^. A growing body of evidence suggests that the gut microbiota, by affecting body metabolism, influences energy balance and glucose metabolism, and changes in its composition are associated with the development of obesity^2^, which is a major risk factor for CRC^6^.

Interestingly, a dynamic but largely stable gut microbiota is beneficial to health^5^. Evidence has shown that microbiota, through the production of reactive sulphur species (RSS)^7^ and short-chain fatty acids (SCFAs)^4^, enhance the host antioxidant capacity. Thus, gut microbiota may be essential in preventing oxidative stress and its related conditions, such as cancer. In addition, previous studies have suggested that gut microbiota modulation may be a novel strategy for CRC prevention and treatment^1^.

Extrapolating from the above, the complex community of microorganisms residing in the GIT (gut microbiota) may serve as a crucial novel strategy for CRC prevention and treatment. Thus, this bibliometric analysis aimed to examine existing research (original research articles) that reported the positive effects of modifying gut microbiota composition with probiotics and/or prebiotics on colorectal cancer, highlighting crucial microbial signatures.

## METHODS

This study employed a systematic data selection and analysis approach to thematically and methodologically review existing studies (original research articles) that report positive effects of altering gut microbiota composition with probiotics and/or prebiotics on colorectal cancer. To ensure a structured and reproducible selection process, a structured data selection approach was implemented to identify and categorise relevant publications systematically. However, it should be noted that this study does not represent a systematic analysis; instead, it applied a structured bibliometric and content analysis to evaluate the positive relationship between gut microbiota and CRC, and research trends in the field. Ethical approval was not required as the analysis was based on previously published data.

### Literature search

**FIGURE 1:**
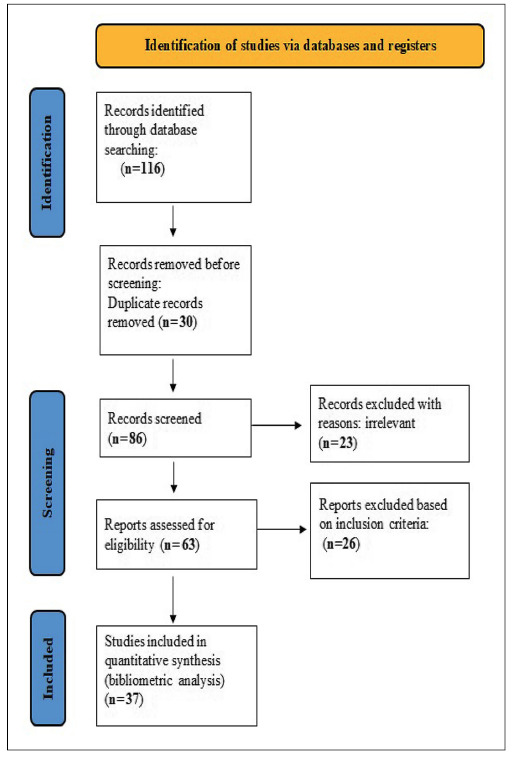
PRISMA flow diagram detailing the literature selection process. This is adapted from Page et al. 2020; Evci and Eser 2025.

This bibliometric analysis was conducted by the PRISMA (Preferred Reporting Items for Systematic Reviews and Meta-Analyses) guidelines^8^ ^-^ ^9^. The literature search was conducted for research articles published online in the English databases Google Scholar and PubMed. In this study, only English peer-reviewed original research articles published between 2020 and 2025 were considered. To search, terms like “Colorectal Cancer” or “Colon Cancer”, “Colorectal Carcinoma” or “Colon Carcinoma”, and “Gut Microbiota”, “Positive Effect Gut Microbiota and Colorectal Cancer” were used. A total of 116 studies were selected and manually reviewed. The PRISMA flow diagram detailing the selection process is provided (Figure 1).

### Inclusion and exclusion criteria

The structured search articles were considered eligible for this study if they met the criteria:

- Peer-reviewed original research articles.
- Studies were in vivo.
- Patients were clinically confirmed with a colon/colorectal tumour.
- Studies reported on gut microbiota composition/profile.
- Studies reported a positive correlation between gut microbiota and colorectal cancer/colon cancer.
- Studies were published between 2020 and 2025.

Studies were excluded if they met the following criteria:

- The same literature(s) was retrieved from the other database (no duplication).
- Reviews, meta-analyses, letters, editorial comments, conference abstracts, case reports, overviews, paediatric articles, unpublished articles, and non-English articles.
- No control groups.
- Studies published before the year 2020.
- The literature(s) was not free.
- Studies reported a negative correlation between gut microbiota and colorectal cancer/colon cancer.

### Literature screening and data extraction

To prevent the inclusion of duplicate papers in this work, the collected articles were imported into Mendeley Desktop (version 1.19.8) to eliminate duplicates. Following that, articles were screened according to the “inclusion and exclusion criteria” of this study and irrelevant studies, such as in vitro studies, were excluded. Finally, data extraction was systematically done and organised in a structured Microsoft Excel spreadsheet considering the following variables:

- The primary study information (year and study location).
- Study subject/experimental unit information (e.g. humans/experimental animals).
- The relationship between gut microbiota and CRC (Positive impact).
- Type of probiotic and/or prebiotic used.
- Sample type (e.g., fecal, intestinal tissue).
- Most and least abundant microbes.
- Identified taxa at the phylum, family, and genus levels

### Data analysis

Data analysis and visualisation were performed using Python 3.11. The analysis pipeline utilised open-source libraries including pandas, matplotlib, and seaborn. The obtained results were presented using bar plots. The following analyses were conducted:

- Annual distribution of publications (2020-2025).
- Geographic distribution by country.
- Classification of studies by type (animal, human, combined) and sample type.
- Most and least abundant microbial taxa reported.
- Taxonomic distribution at the phylum, family, and genus levels.

## RESULTS

### Study selection

The literature search and selection process are illustrated in FIGURE 1. In the initial systematic search, we obtained a total of 116 studies from English electronic databases, namely Google Scholar and PubMed. Before screening, we excluded 30 duplicate articles. The remaining 86 articles were screened based on their titles and abstracts, resulting in the exclusion of 23 additional articles. Consequently, 63 articles were deemed eligible for assessment. After reviewing the full texts of these 63 articles, we excluded 26 based on the study’s inclusion criteria. Ultimately, 37 research articles met the study criteria and were included in this bibliometric analysis.

### Annual distribution of publications

**FIGURE 2:**
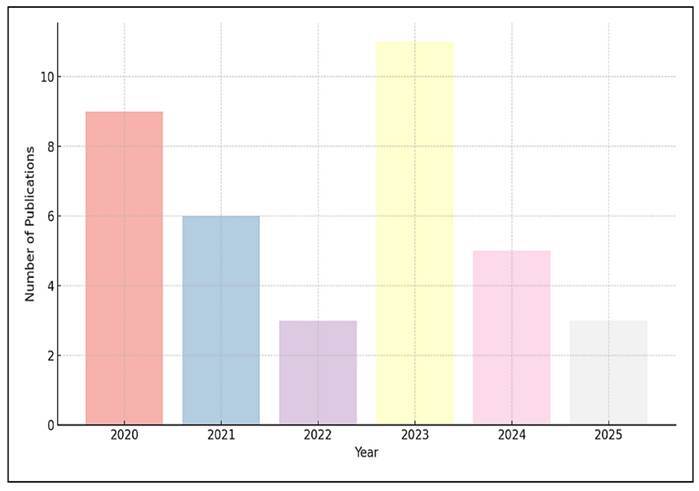
The annual distribution of publications.

The annual distribution of publications on probiotics and prebiotics affecting gut microbiota from 2020 to 2025 is shown in Figure 2. Between 2020 and 2023, the number of publications increased progressively, rising from 2020 (n=6, 16.2%) to 2022 (n=9, 24.3%) to 2023 (n=11, 29.7%). This trend suggests a growing academic interest in interventions that aim to modify the microbiota. However, it is interesting to note that the year 2021 also exhibited a modest decline in publications (n=3, 8.1%), which may be indicative of transient fluctuations in research output or reporting. Also, a notable decrease in publication volume is observed in the following years, with only 5 publications in 2024 (13.5%) and 3 in 2025 (8.1%). This decline may be attributed to several factors: a possible stabilisation of research output in the field, a shift toward more specialised or mechanistic studies, or a time lag in indexing recent literature, especially for 2025. Notably, the year 2025 saw a lower number of publications, with just three, which could reflect a temporary dip in research activity.

### Distribution of studies by country

**FIGURE 3:**
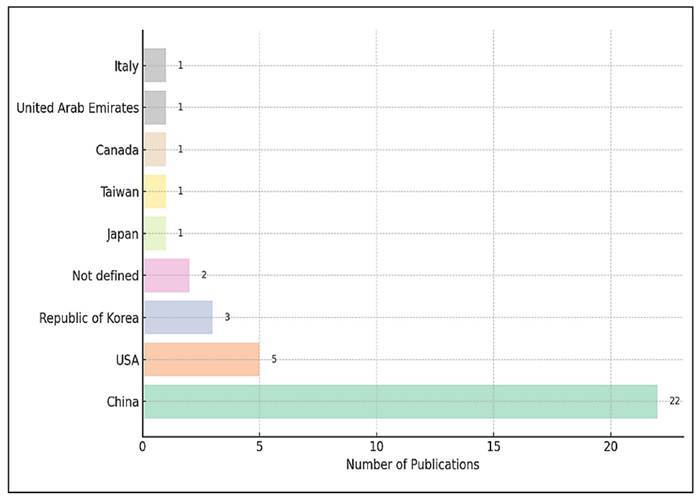
Distribution of studies by country.

The geographical distribution of studies examining the effects of probiotics and/or prebiotics on gut microbiota is illustrated in Figure 3. A clear dominance by a few countries is evident, reflecting regional trends in research productivity and investment in microbiome science.

China is the leading contributor with 22 publications, making up a significant portion of the total dataset (59.5%). This strong position can be attributed to the country’s growing scientific output, increased governmental support for health-related biotechnology, and a strategic focus on gut microbiota research in recent years. China’s interest in functional foods, traditional medicine, and dietary modulation likely supports this trend.

The United States ranks second with 5 publications (13.5%), reflecting its strong research infrastructure and its established contributions to nutrition and microbiome science. However, its output on this specific topic seems modest compared to China, which may indicate that the U.S. has more diversified research priorities.

Several other countries, including South Korea (3 publications, 8.1%), Japan, Taiwan, Canada, the United Arab Emirates, and Italy (each contributing 1 publication, 2.7% respectively). This varied involvement indicates a rising, yet still limited, global interest in probiotic and/or prebiotic-related microbiota research beyond the leading nations.

### Study type distribution based on experimental units

**FIGURE 4:**
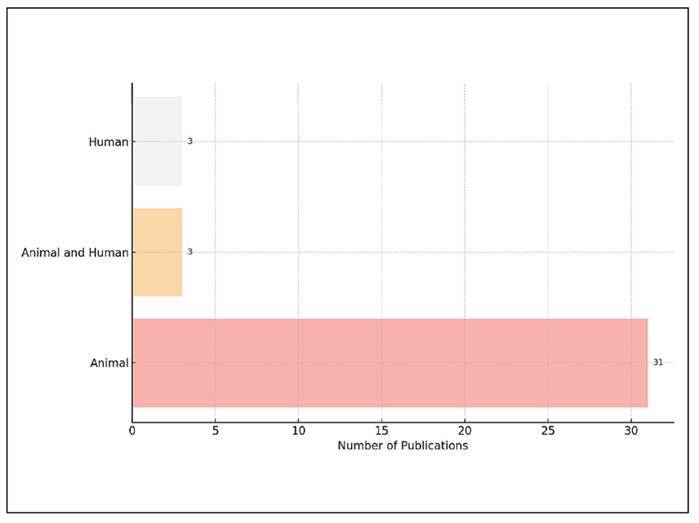
Distribution based on the type of experimental units.

Figure 4 shows the classification of the included studies based on their subject units. An analysis of the distribution of study types among the 37 unique articles revealed a strong predominance of animal-based research. Specifically, 31 studies (83.8%) focused exclusively on animals, indicating a significant reliance on preclinical models in probiotic and prebiotic research. In contrast, only 3 articles (8.1%) were based on human subjects, and another 3 articles (8.1%) incorporated data from both animal and human sources. This distribution highlights a significant gap between experimental research and clinical application in this field. The low percentage of studies involving humans and translational research indicates that, despite the growing interest in microbiome modulation, the scientific literature continues to depend heavily on animal models for investigating gut microbial dynamics and host responses. The lack of combined studies involving both animals and humans highlights the limited efforts to verify findings from animal research in human populations. This validation is crucial for connecting laboratory results with clinical applications. These observations underscore the necessity for future research to broaden the representation of human cohorts and to encourage integrative designs that allow for comparisons across species. Such methods could improve the external validity of microbiome research and aid in the development of targeted dietary or therapeutic interventions that have proven effective in human subjects.

### Biological sample types

**FIGURE 5:**
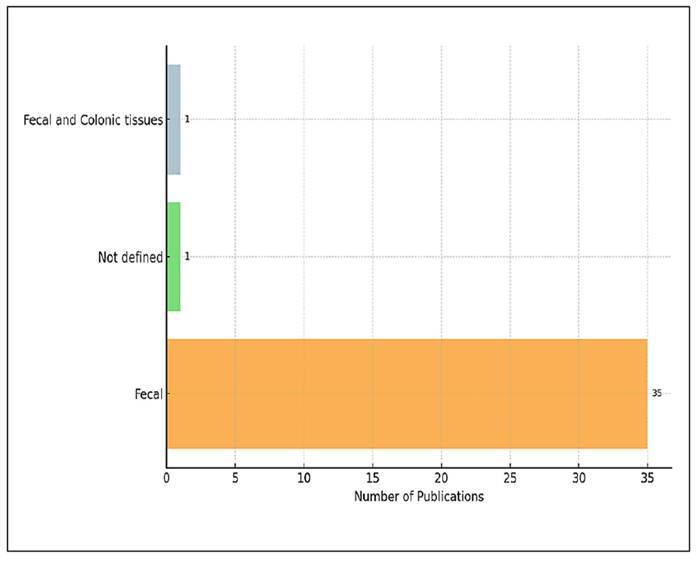
Types of biological samples used for microbiota profiling.

Figure 5 illustrates the distribution of biological sample types used in studies that evaluate the impact of probiotics and/or prebiotics on gut microbiota composition. Among the 37 unique studies included in this analysis, a clear methodological trend was observed regarding the biological sample types utilised for microbiota profiling. Faecal samples were used in 35 studies (94.6%), making them the most commonly adopted sample type. One study (2.7%) utilised a combination of faecal and colonic tissue samples, while another study (2.7%) did not specify the sample type used. The predominance of faecal sampling highlights its methodological advantages, including non-invasiveness, ease of collection, and suitability for both animal and human research. The limited use of colonic tissue samples underscores the challenges associated with invasive procedures, particularly in human studies.

### Classification of studies based on mode of microbiota modification

**FIGURE 6:**
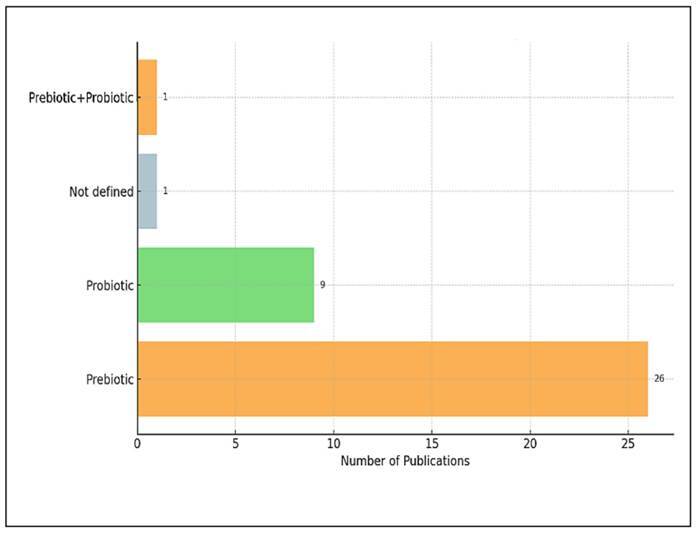
Depicts the additive used (probiotic and/or prebiotic) in studies.

Figure 6 presents the classification of 37 unique studies based on their use of probiotics, prebiotics, or a combination of both. The data indicate a significant predominance of prebiotic-focused interventions in the literature. Among the included studies, 26 studies (70.3%) utilised prebiotics exclusively, while 9 studies (24.3%) concentrated solely on probiotics. Only 1 study (2.7%) employed a combined approach that incorporated both probiotics and prebiotics, known as a synbiotic design. Additionally, another study (2.7%) did not clearly define the type of intervention used. This distribution suggests that prebiotics have been more frequently researched, likely due to their easier formulation, more consistent regulatory status, and broader dietary applications. In contrast, the studies focusing solely on probiotics were less common, and the use of combined approaches remains underrepresented, even with the growing interest in synbiotic therapies. The limited number of studies investigating combined interventions may reflect challenges related to study design, safety concerns, or difficulties in understanding the underlying mechanisms. Nonetheless, this trend highlights a significant opportunity for future research to explore the synergistic effects of probiotics and prebiotics on gut microbiota composition and overall host health outcomes.

### Types of probiotics used in studies

**FIGURE 7:**
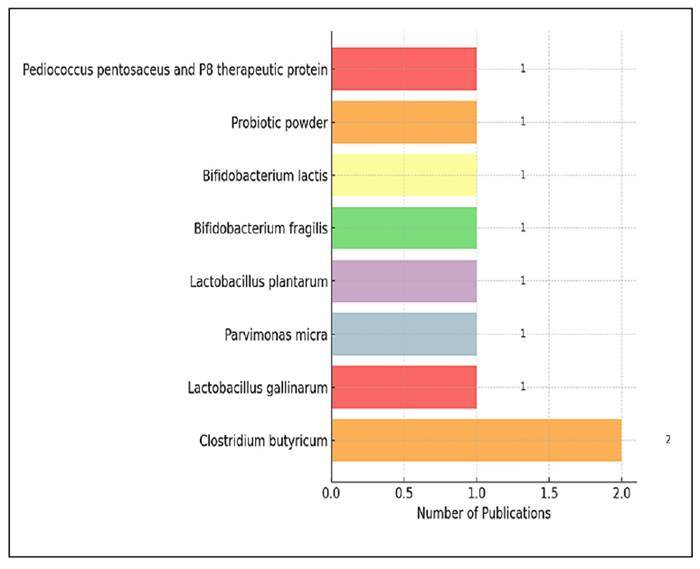
Classification of probiotic types used in studies.

Figure 7 illustrates the classification of probiotics used in the studies analysed. Among the 9 studies that used probiotics as the sole intervention, specific probiotic strains were reported in seven studies. *Clostridium butyricum* was the most frequently used species, appearing in two studies (22.2%). Each of the following probiotic types was reported in one study (11.1% each): *lactobacillus gallinarum*, *Lactobacillus plantarum*, *Parvimonas micra*, *Bifidobacterium fragilis*, *Bifidobacterium lactis*, *Pediococcus pentosaceus* and an undefined “probiotic powder.” In addition to *Clostridium butyricum*, *Lactobacillus spp*. (*Lactobacillus gallinarum* and *Lactobacillus plantarum*) and *Bifidobacterium spp*. (*Bifidobacterium fragilis* and *Bifidobacterium lactis*) were reported in two studies each, making them also the most frequently used species.

### Types of prebiotics used in studies

**FIGURE 8:**
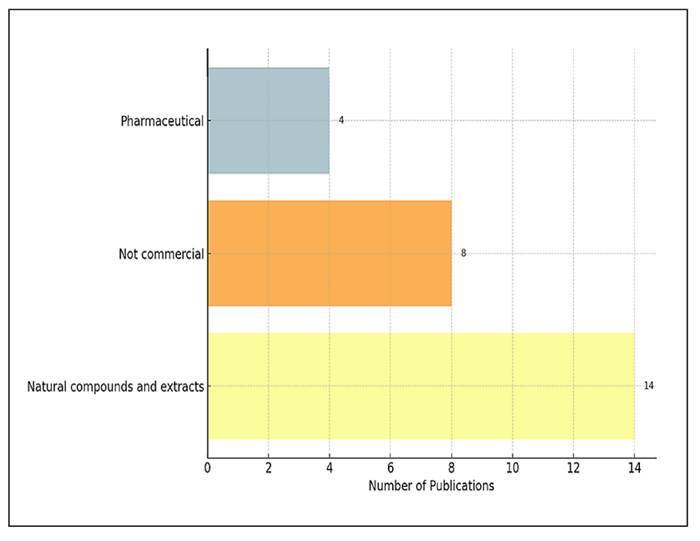
Classification of prebiotic types used in studies.

Figure 8 illustrates the distribution of prebiotic classifications among the studies included in this analysis. A significant finding is the clear dominance of the “Natural compounds and extracts” category, which comprises more than half of the classified studies (n=14, 53.8%). This category encompasses a diverse array of plant-based substances, herbal decoctions, polysaccharides, and other bioactive dietary components. In contrast, a smaller group of studies utilised “Not commercial” prebiotics (n=8, 30.8%), referring to experimental or naturally derived substances that are not associated with any specific commercial formulation. Pharmaceutical agents, such as antibiotics, antidiabetic drugs, or inhibitors, were examined in 4 studies (15.4%). These findings highlight a relatively high level of reporting rigour for prebiotic interventions.

### Frequently identified microbial phyla

**FIGURE 9:**
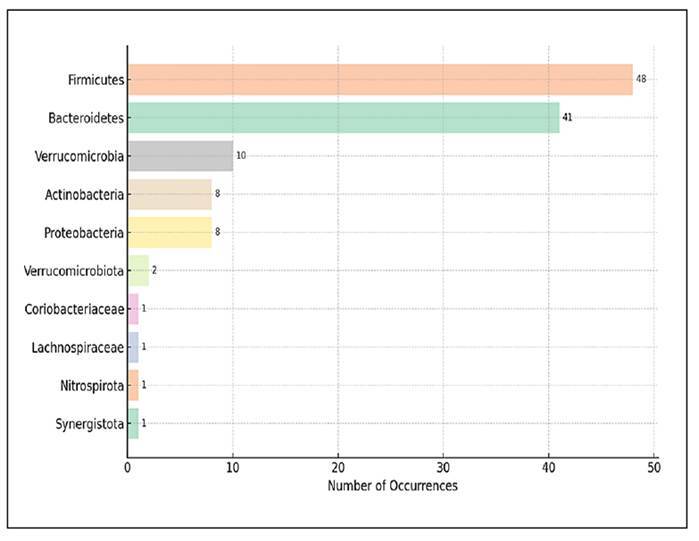
Top 10 identified microbial phyla in studies.

Figure 9 summarises the 10 most frequently identified microbial phyla across studies examining the impact of probiotics and/or prebiotics on gut microbiota composition. The taxonomic data were standardised to account for synonymous classifications (e.g., *Verrucomicrobia* and *Verrucomicrobiota* were combined). The phylum *Firmicutes* was the most commonly reported, appearing in 48 studies. This dominance is consistent with its known abundance in the gastrointestinal tract and its central role in fermenting dietary fibres, producing short-chain fatty acids, and influencing host energy balance. 

Following *Firmicutes*, the phyla *Bacteroidetes* (in 41 studies) and *Verrucomicrobia/Verrucomicrobiota* (appeared in 12 studies, if merged) were also frequently reported, reflecting their well-established importance in gut ecology and health. Other phyla, such as *Proteobacteria*, *Actinobacteria*, and *Nitrospirota*, were observed less frequently, suggesting more targeted or niche-focused investigations. 

### Frequently identified microbial families

**FIGURE 10:**
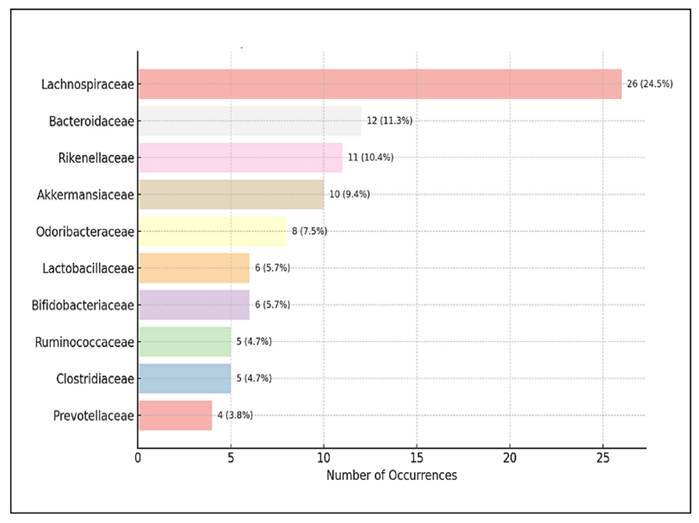
The top 10 identified microbial families in the analysed studies.

Figure 10 shows the ten most commonly identified microbial families in studies exploring the effects of probiotics and/or prebiotics on gut microbiota. The data highlight a prevalence of findings related to bacterial families that are typically associated with gut health, energy metabolism, and interactions between hosts and microbes. The most frequently reported family is *Lactobacillaceae*, accounting for 24.5% (n=26), which aligns with the widespread use of *Lactobacillus species* in probiotic products. Members of this family are well-known for their roles in maintaining the integrity of the mucosal barrier, modulating immune responses, and producing antimicrobial compounds like bacteriocins. *Bacteroidaceae* was the second most prevalent, accounting for 11.3% (n=12).

Following closely in frequency are *Rikenellaceae* (10.4%, n=11) and *Akkermansiaceae* (9.4%, n=10). *Akkermansia muciniphila*, a key representative of the *Akkermansiaceae* family, has gained significant attention for its role in mucin degradation and its potential anti-obesity and anti-diabetic properties. Other families, including *Odoribacteraceae* (7.5%), *Lactobacillaceae* and *Bifidobacteriaceae* (5.7% each), were also present at moderate frequencies and are commonly associated with probiotic functions.

*Ruminococcaceae* and *Clostridiaceae* each accounted for 4.7%, along with *Prevotellaceae* (3.8%) constituted the top ten, thereby emphasising the taxonomic diversity of the microbial communities evaluated across the included studies. The distribution of taxa reflects both core gut microbiota members and taxa of emerging interest in the field of microbiome research.

### Frequently identified microbial genera

**FIGURE 11:**
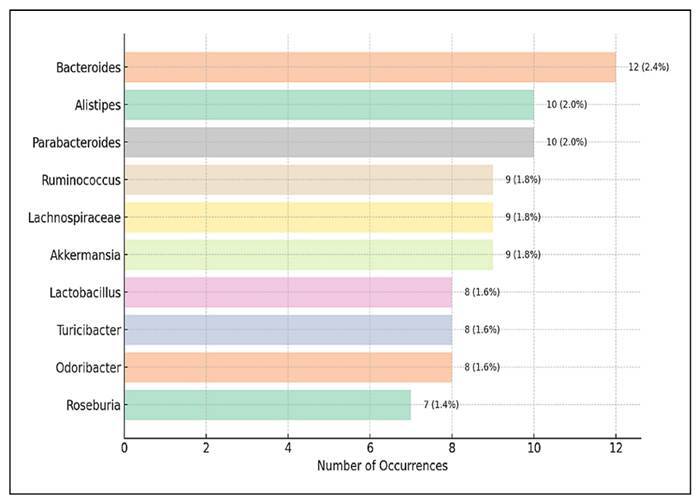
The top 10 identified microbial genera in the analysed studies.

Figure 11 shows the ten most frequently identified microbial genera in studies examining the effects of probiotics and/or prebiotics on gut microbiota. Unlike ranking dominance or suppression within individual studies, this distribution reflects the overall frequency of each genus reported in the literature, providing insights into the most commonly targeted or observed microbial groups.

The genus *Bacteroides* ranked highest, appearing in 2.4% (n=12). As a core component of the human gut microbiota, *Bacteroides species* play critical roles in breaking down complex polysaccharides, modulating the host immune system, and producing short-chain fatty acids (SCFAs). Their prevalence in scientific literature may be due to their sensitivity to dietary interventions and their role as indicators of microbiota functionality and composition.

*Alistipes* and *Parabacteroides*, both members of the *Bacteroidetes* phylum, were also frequently reported in 2.0% (n=10) of entries. *Alistipes* is increasingly associated with stress, inflammation, and disease states when dysregulated, while *Parabacteroides* has been studied for its potential anti-inflammatory and metabolic roles. Their repeated identification suggests a dual role in both beneficial and dysbiotic gut environments, depending on the context.

Key members of the *Firmicutes* phylum, such as *Lachnospiraceae* and *Ruminococcus*, were also prominently featured, each appeared in 1.8% (n=9) of enteries. These genera are major producers of butyrate, essential for maintaining epithelial integrity and modulating host metabolism. The presence of *Akkermansia* (1.8%, n=9) of entries, particularly the species *A. muciniphila*, further highlights its emerging significance, as it is linked to mucin turnover and metabolic health benefits.

Other frequently reported genera include *Odoribacter* and *Turicibacter* (1.6%, n 8 each), which are typically categorised as low-abundance but functionally significant taxa. These genera have been implicated in various host physiological processes, especially lipid metabolism and gut motility. Additionally, *Roseburia* is noted in 1.4% (n=7) of entries, a prominent butyrate-producing genus, has been recognised for its role in anti-inflammatory pathways and glucose homeostasis. Lastly, *Lactobacillus* appeared in 1.6% (n=8) of entries, a well-established and widely used probiotic genus, which is commonly included in commercial formulations due to its ability to restore microbial balance and support mucosal immune function.

### Most abundant microorganisms in individual studies

**FIGURE 12:**
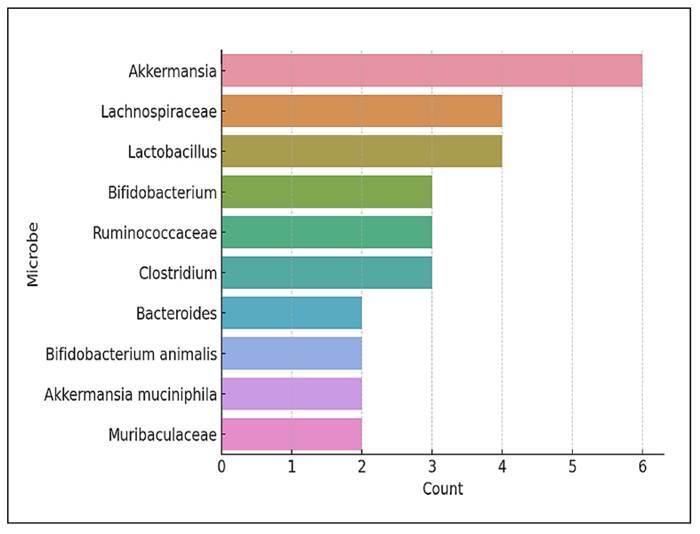
Top 10 most-abundant microorganisms in individual studies.

Figure 12 presents the microbial taxa most frequently reported as the dominant (most abundant) organisms within individual studies. Instead of showing overall abundance across pooled datasets, this graph highlights the specific taxa recognised as the most prominent in relative abundance for each study context. The genus *Akkermansia* was the most frequently cited as the dominant microbe, being reported as the highest-abundance taxon in six separate studies. This aligns with the growing interest in *Akkermansia muciniphila*, due to its mucin-degrading properties and beneficial metabolic associations, including improved glucose tolerance and anti-inflammatory effects.

Other genera or families commonly identified as dominant in studies included *Lachnospiraceae*, *Lactobacillus*, and *Bifidobacterium*. These taxa are well-known for their roles in the production of short-chain fatty acids, maintenance of gut barrier integrity, and regulation of the host immune system. The presence of *Ruminococcaceae*, *Clostridium*, and *Bacteroides* further emphasises their ecological importance and responsiveness to prebiotic or probiotic interventions.

Interestingly, taxa such as *Bifidobacterium animalis*, *Akkermansia muciniphila*, and *Muribaculaceae*, while not universally dominant, were noted as peak-abundance organisms within individual studies. This indicates their potential for context-dependent expansion under certain experimental conditions.

### Least abundant microorganisms in individual studies

**FIGURE 13:**
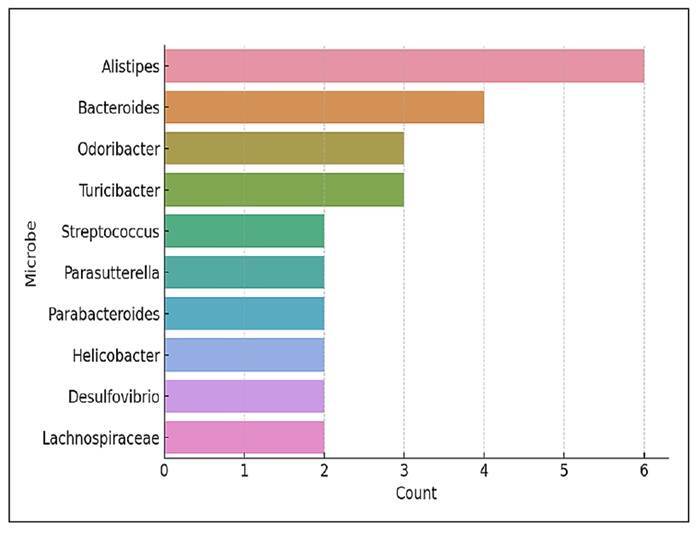
Top 10 low-abundant microorganisms in individual studies.

Figure 13 displays the microbial taxa that were most frequently reported as having low abundance in individual studies. It is important to note that this does not indicate overall low abundance across all samples; rather, it reflects which taxa were identified as the least dominant within specific experimental or observational contexts. The genus *Alistipes* was the most commonly reported low-abundance taxon, identified as the least represented organism in six studies. Although *Alistipes* is a common gut commensal, its low abundance may indicate sensitivity to specific probiotic or prebiotic interventions or associations with compromised ecological niches within the microbiota. 

Other taxa frequently identified as having low abundance include *Bacteroides*, *Odoribacter*, and *Turicibacter*. Although these genera are often abundant in healthy individuals, they may experience significant reductions under certain dietary conditions, during inflammation-driven dysbiosis, or as a result of targeted microbial modulation strategies.

Additionally, *Streptococcus*, *Helicobacter*, and *Desulfovibrio* appeared among the least abundant taxa. *Helicobacter* and *Desulfovibrio* are particularly noteworthy due to their potential pathogenicity and their links to mucosal inflammation and sulfur metabolism, respectively. Their low abundance in many studies may reflect intentional suppression or baseline scarcity in healthy models. 

Finally, the presence of *Lachnospiraceae* and *Parabacteroides* on this list, despite their common identification as beneficial or butyrate-producing organisms, suggests context-dependent depletion, such as during fibre-restricted diets or high-fat feeding regimens.

## DISCUSSION

The primary risk factors for CRC include advanced age and genetic and environmental factors^1^. Despite this, there has been significant interest in the role of gut microbiota in the development and progression of CRC. This interest is probably a result of the high load of microbes in the colon and the implication of the gut microbiota in carcinogenesis. The gut microbiota is the largest and most influential microecosystem in the body and plays a pivotal role in maintaining overall health^10^. Currently, standard therapies for CRC include immunotherapy, chemoradiotherapy, and surgery, all of which are associated with recurrence, metastasis, and poor prognosis^10^. This highlights the need for alternative treatment strategies. One promising treatment approach is the modulation of the gut microbiota using natural compounds or extracts, such as Huangqin Decoction^10^. Research has shown that by modulating the gut microbiota, the therapeutic impact of 5-fluorouracil (5-FU), a drug commonly used to treat gastrointestinal tumours, is enhanced while mitigating its side effects. Thus, the modulation of gut microbiota plays an essential role in the treatment of CRC and has been recognised as a promising mechanism for the treatment of CRC^11^.

In this bibliometric analysis, 37 publications were examined. China emerged as the leading contributor, with 22 publications accounting for 59.5% of the dataset. This prominence can be attributed to the country’s increasing investment in scientific research and its interest in traditional medicine. In this study, a marked preference for using prebiotics and/or natural compounds (such as extracts) to modulate the gut microbiota was observed, as most of the publications from China focused on Traditional Chinese Medicine^10^ ^-^ ^20^. Among the 37 studies analysed, 70.3% focused solely on prebiotics, 24.3% used probiotics, and only 2.7% adopted a combined approach (probiotics and prebiotics). This indicates that the preferred mode of modifying the gut microbiota is distinctly dominated by prebiotics, products derived from dietary fibres, or plant compounds that are easier to integrate into functional foods^21^ ^,^ ^22^.

In contrast, the use of probiotics, which involves the administration of live microorganisms, faces various challenges, such as species-specific efficacy, variability in responses among individuals, and difficulties in transportation and storage conditions^23^ ^,^ ^24^. These challenges may have led to a lesser representation of probiotic studies in the literature. Although the combined use of prebiotics and probiotics could theoretically exhibit synergistic effects by enhancing microbial viability and supporting targeted metabolic activities, factors such as the selection of compatible probiotic strains and appropriate prebiotic substrates complicate the use of this approach. Nevertheless, the use of well-designed combinations can contribute to the development of more effective therapeutic strategies for managing diseases associated with dysbiosis^25^.

The classification of prebiotic types used in studies revealed a distinct trend towards natural compounds and plant-derived bioactive substances. More than half of the prebiotic interventions (53.8%) consisted of natural extracts, herbal ingredients, and dietary polysaccharides. This reflects the interest in the effects of traditional medicine and functional foods on the gut microbiota, particularly in Asian countries^26^.

Considering the annual distribution of publications, this study reports a decline in publications on this subject, as 2025 contributed only three publications to the dataset (8.1%). This could mean that research on the subject is shifting to more specialised studies, such as the use of non-commercial or experimental prebiotics. The use of non-commercial or experimental prebiotics (30.8%) indicates that microbiota research is still in its exploratory phase. This group includes new oligosaccharides or unidentified fibre complexes, and the prebiotic potential of these components has not yet been definitively established. While this provides scientific innovation, it can also lead to variability in the results due to differences in content and dosage. The analysis of probiotic species used in studies has been noteworthy in terms of microbial diversity, but it has revealed that the number of probiotic-focused interventions remains limited. *Clostridium butyricum* was the most commonly used species, appearing in two of the nine studies (22.2%). Additionally, species from the *Lactobacillus* and *Bifidobacterium* genera were included in two studies each, demonstrating that these groups are important in probiotic research^24^ ^,^ ^26^.

Xue et al.^17^ reported *Firmicutes* and *Bacteroidetes* as the most dominant phyla, consistent with the trend of a higher frequency of *Firmicutes*, followed by *Bacteroidetes*, observed in the studies included in this analysis. This trend is likely because these phyla are the most abundant microbes in the gut, as species from the phyla *Bacteroidetes* and *Firmicutes* account for over 90% of the gut microbiota^10^. At the genus level, *Akkermansia spp*. was the most frequently cited dominant microbe (most abundant). This is probably due to its ability to inhibit the occurrence of colonic tumours and ensure intestinal epithelial barrier integrity by exhibiting protective effects to reduce mucosal inflammation that may have been induced by colitis or CRC^11^. Following *Akkermansia*, *Lachnospiraceae* was the most abundant beneficial bacterium in a healthy gut^10^.

When evaluated from the perspective of experimental design, animal models have stood out in studies examining the effects of probiotics and prebiotics on the gut microbiota and host physiology. Of the 37 articles assessed, 83.8% were based solely on animal subjects, 8.1% were based on human subjects, and 8.1% included both human and animal data. Although experimental animal models are often preferred because of their relatively low cost, ease of manipulation, and controllable experimental environments, their direct applicability to human physiology presents a significant limitation^28^ ^,^ ^29^. Differences in metabolic pathways, immune responses, and microbial loads among species make it difficult to translate data obtained from experimental models to humans directly^27^ ^-^ ^28^ ^,^ ^30^. Therefore, the increasing importance of studies that include both animal and human data is crucial for validating the findings obtained and establishing a solid foundation for clinical applications. Walter et al.^31^ stated that inter-species comparative designs are critical for understanding independent microbiome responses and optimising interventions specific to human health.

In studies evaluating the effects of probiotics and/or prebiotics on the gut microbiota, a distinct methodological trend towards using faecal samples appears. The fact that faecal samples were used in 94.6% of the 37 analysed studies shows that this sample type has become the standard for microbiota profiling. This finding is consistent with existing trends in the literature, as faecal sampling is considered a practical, noninvasive, and repeatable method for both human and animal studies^32^ ^,^ ^33^.

## CONCLUSION

In conclusion, the relationship between gut microbiota and colorectal cancer (CRC) highlights the complexity and significance of microbial communities in health and disease. While certain taxa demonstrate context-dependent abundance variations, the modulation of gut microbiota through natural compounds, particularly prebiotics, shows promise as a complementary strategy in CRC treatment. The predominance of studies focused on prebiotics, especially from China, indicates a growing interest in leveraging traditional medicine and dietary approaches for enhancing therapeutic outcomes. While challenges remain in the use of probiotics, exploring the synergistic effects of combined prebiotic and probiotic interventions could pave the way for more effective CRC management strategies. However, further research is essential to deepen our understanding of gut microbiota dynamics and to optimise therapeutic approaches for CRC patients.

## Data Availability

Data-available-upon-request
